# Changes in Haematological Parameters and Lipid Profiles in Diabetes Mellitus: A Literature Review

**DOI:** 10.7759/cureus.64201

**Published:** 2024-07-09

**Authors:** Jovita I Mbah, Phillip T Bwititi, Prajwal Gyawali, Ezekiel U Nwose

**Affiliations:** 1 School of Health and Medical Sciences, University of Southern Queensland, Toowoomba, AUS; 2 School of Dentistry and Medical Sciences, Charles Sturt University, Bathurst, AUS; 3 Department of Public and Community Health, Novena University, Ogume, NGA

**Keywords:** type 2 diabetes mellitus, haematology, dyslipidaemia, correlation, cardiovascular complications

## Abstract

Diabetes mellitus is a metabolic disorder characterized by elevated blood glucose that has sequelae on cellular, haematological, and metabolic parameters, including lipid profile disturbed homeostasis, which manifest in alterations in haematological parameters and lipid profiles. These changes in haematological parameters and lipid profiles have been reported by previous research; however, the pattern of these changes and their correlation have not been elucidated. This review aims to assess these changes and investigate the degree of correlation between haematological parameters and lipid profiles in patients with type 2 diabetes mellitus (T2DM).

The method adopted was a traditional review approach that included a narrative of concepts and a critical assessment of a few selected articles. Findings highlight that haematological parameters and lipid profiles show varied alterations and correlations in T2DM. For instance, statistical significances at p < 0.05 are reported for WBC count (r = -0.75) showing negative correlations (p < 0.001), where RBC count (r = 0.56) showed correlation with high-density lipoprotein cholesterol (HDLC), whereas anaemia (packed cell volume: r = -0.51) and RBC indices (mean corpuscular volume: r = -0.75; mean corpuscular haemoglobin: r = -089) show negative correlations with total cholesterol (TC). The specific haematological parameters, namely, RBC and WBC with differential and platelet counts, as well as indices, showed varied changes and correlation with lipid profiles, namely, HDLC, low-density lipoprotein cholesterol, TC, and triglyceride, in the six reviewed articles.

Diabetes is characterized by changes in haematological parameters and lipid profiles. A better understanding of the negative and positive correlating changes could be utilized in routine evaluation of subjects with prediabetes as well as managing complications in diabetes. Correlation between haematological parameters and lipid profiles over the course of diabetes progression using HbA1c as an index of glucose control is necessary for additional empirical data and updates.

## Introduction and background

Diabetes mellitus is a chronic condition that occurs when raised levels of blood glucose occur. It is common knowledge that this occurrence is due to insulin resistance (i.e. type 2 diabetes mellitus, T2DM) or deficiency (i.e. type 1 diabetes mellitus, T1DM). It is also known that multiple other factors including lipidomics as well as cellular progenitors such as granulocytes and macrophages are implicated in diabetes pathogenesis [1]. Lipid abnormalities are common features of diabetes either due to insulin resistance or deficiency affecting major enzymes and pathways in lipid metabolism [2]. Elevated blood glucose seen in diabetes is closely associated with dyslipidaemia, but it is unclear if dyslipidaemia plays any causal relationship in T2DM [3]. Dyslipidaemia in diabetes is usually characterized by clusters of interrelated plasma lipid and lipoprotein abnormalities. This includes reduced levels of high-density lipoprotein cholesterol (HDLC), elevated triglycerides (TG), and low-density lipoprotein cholesterol (LDLC) [4], and raised levels of total cholesterol (TC). These raised levels of TC, TG, and LDLC and reduced levels of HDLC are the currently accessible clinical lipidomics or lipid profiles. These parameters are known predictors of coronary heart disease (CHD) [5].

Excess body mass, characterized in part by excess adipose tissue, is found to be responsible for metabolic disorders that lead to T2DM and hyperlipidemia, which in turn give rise to the accelerated development of arteriosclerosis [6]. This report also showed that subjects with diabetes had twice as much blood glucose as control subjects, and this high blood glucose correlated significantly and positively with low-density lipoprotein (LDL) (r = 0.34, p ≤ 0.05). Lipid ratios are also known to be associated with complications of diabetes, such as cardiovascular disease (CVD). The accumulation of numerous cardiovascular risk factors, including obesity and dyslipidaemia, is the basic phenomenon of insulin resistance. Hyperinsulinaemia, which is an important feature of insulin resistance, is also regarded as a risk factor for CVD [7].

Chronic inflammation, which is an attribute of insulin resistance, is closely associated with diabetes mellitus and atherosclerosis. In diabetes, there is a tendency for multiple factors, such as hyperglycaemia and dyslipidaemia, to be at play. There is also the knowledge that inflammatory molecules such as calprotectin and cytokines are at play, as well as the fact that these molecules interact with bone marrow receptors for advanced glycation products on common myeloid progenitors and impact granulocyte-macrophage progenitors, which in turn influence both circulating WBC and atherosclerotic plaque formation [1]. Perhaps what is uncommonly known is a gap in knowledge and practice whereby evaluation of intricate blood cell indices and possible correlation with currently assessed lipidomics have yet to be part of the clinical protocol.

Chronic inflammation is linked with an increase in the level of cytokines as well as an alteration in haematological parameters, notably leukocytes, and the process of erythropoiesis. Poor glycaemic control can lead to several complications, such as nephropathy, retinopathy, neuropathy, and oxidative stress, leading to an alteration in the morphology and structure of blood cells, which in turn results in haematological changes [8]. The achievement of good glycemic control is of utmost importance in the management of diabetes. Predictors of poor glycaemic control include rural residence, age of patients, duration of diabetes, time since diagnosis, and drug regime or insulin treatment [9]. The changes due to poor glycaemia have been reported to affect RBC, WBC, platelets, and coagulation factors, and these are directly related to diabetes mellitus [10]. According to Antwi-Baffour et al., differential WBC showed raised levels of basophils, eosinophils, and neutrophils, while monocytes showed no changes in diabetic patients [10]. This report, however, contradicted the findings of Madjid et al., whereby raised eosinophils, neutrophils, and monocytes were reported in diabetes [11]. Therefore, there is credible evidence that leukocytosis affects CVD, including atherosclerosis and CHD, through multiple pathologic mechanisms that mediate inflammation, cause proteolytic and oxidative damage to endothelial cells, plug the microvasculature, and induce hypercoagulability [1,11]. Further studies would be necessary for these reports.

T2DM, as part of the metabolic syndrome (MetS), is a growing public health concern with high morbidity and mortality. Diabetes comorbidity with other components of MetS constitutes a major risk factor for CVD [12]. There is up to a 90.6% prevalence of MetS, and a normal obesity index is rare among individuals living with T2DM, with a higher percentage in females compared to males [13]. As the mortality of MetS among diabetic patients is increasing, identifying risk factors that predispose diabetic patients to MetS may create an opportunity to change their lifestyle and develop therapeutic measures where necessary to prevent more complications [14].

Statement of the problem

Critical to the management of diabetes is dyslipidaemia, which needs to be managed to reduce the incidence of CVD. Although dietary intervention and exercise can in certain cases reduce diabetic dyslipidaemia, many cases will require pharmacological intervention such as antioxidants and statins [15]. Given that in diabetes, haematological alterations are associated with increased myelopoiesis and blood cell dysfunction [1,11], while dyslipidaemia is usually found in diabetes [3-5], the knowledge of the interaction/association between haematological parameters and lipid profiles could offer a new approach in the management of diabetes.

Objectives

Therefore, the overall objective of this review is to elucidate the concordance of haematological parameters and lipid profiles in diabetes. Specifically, both conceptual and empirical reviews are done for the following specific objectives: (1) The review of concepts is to narrate the available evidence regarding changes in haematological parameters and lipid profiles in diabetes mellitus. (2) The empirical review of correlations is to explore available evidence on how serum lipid profile patterns are correlated with different haematological parameters.

Justification of importance

Regular laboratory monitoring of lipids, but not haematological indices, is specifically recommended in clinical guidelines for diabetes management. Knowledge of the correlation between haematological parameters and lipid profiles could offer a rationale to consider the recommendation of haematology in a new approach for the improved management of diabetes. While the development of dyslipidaemia constitutes a risk of future diabetes complications, understanding the relationship between serum lipid patterns and different haematological parameters is useful in clinical practice to identify haematological evaluations in preventive programs for diabetes and related complications. This is important given that routine haematology is cheaper and more accessible than lipid profiles, especially in rural and remote health as well as low- and middle-income countries (LMIC).

## Review

Review methods

**Figure 1:**
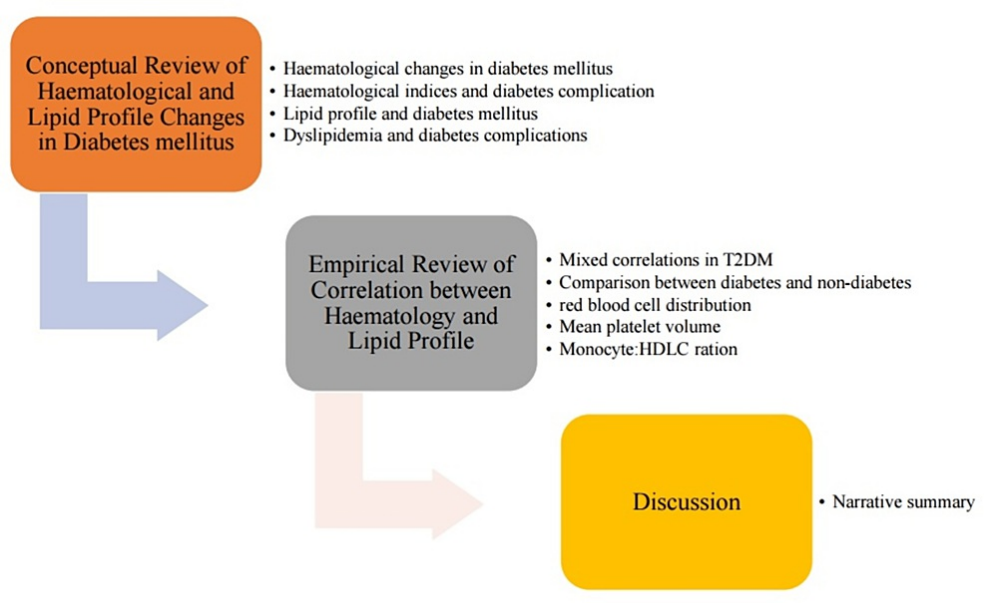
Graphical outlay of the review T2DM: type 2 diabetes mellitus, HDLC: high-density lipoprotein cholesterol

The graphical overview of the review is presented to highlight the two sections and their subsections (Figure 1). The first section is the narrative review of the concepts of haematological parameters and lipid profiles in diabetes, which followed the Scale for Assessment of Narrative Review Articles (SANRA) [16]. The second section was a critical review of the correlation between haematological parameters and lipid profiles in diabetes, which adopted the Preferred Reporting Items for Systematic Reviews and Meta-Analyses (PRISMA) flowchart [17]. The criteria for literature articles were studies involving participants living with diabetes, and data analysis necessarily included these laboratory results. The third section is the discussion.

Conceptual review of haematological parameter and lipid profile changes in diabetes mellitus

Research papers on ‘haematological parameters in diabetes mellitus’ and ‘lipid profile changes in diabetes mellitus’ were separately searched for, starting with Google Scholar. Relevant articles that are indexed on PubMed were discretionally selected for review. Further, references within articles were reviewed by titles, and relevant ones were selected for additional information.

Haematological Changes in Diabetes Mellitus

Diabetes is directly linked to changes in haematopoietic activities and, therefore, affects RBC, WBC, and coagulation factors [18]. This alteration of haematopoiesis compromises the functions of the bone marrow, producing a stem cell niche-dependent defect in haematopoietic stem cell mobilization resulting in a decrease in RBC count [19]. Sustained hyperglycaemia leads to increased production of reactive oxygen species associated with endothelial tissue damage, RBC dysfunction, and inflammation [20]. Anaemia is common and can be seen in diabetic patients, usually occurring earlier and to a higher degree in patients with diabetic nephropathy [21]. This factor of anaemia could be confounded by chronic kidney disease, chronic inflammation, and higher levels of oxidative stress common in a diabetic environment, all of which can reduce RBC’s survival, resulting in variation in erythrocyte size and a lower count [22].

There has been renewed interest in haematological parameters such as WBC count, red cell distribution width (RDW), mean platelet volume (MPV), platelet distribution width (PDW), and platelet count as predictors of endothelial dysfunction and inflammation [23]. In a diabetic environment, WBC can be stimulated by advanced glycation end products, oxidative stress, and cytokines. Activated WBCs release different kinds of cytokines and transcription factors, which play a fundamental role in inflammation [24]. Platelet count and MPV are indicators of thrombotic events and risk factors for diabetic microvascular complications. Platelet count was higher in diabetics compared to normal controls [25].

It has been reported that although the mean platelet count was higher in patients with diabetes compared to the control group, the difference was not statistically significant [26]. However, there are contradictory reports. A study reported that platelet count was significantly decreased in diabetics compared to controls, but platelet count did not show statistical significance in diabetic patients with complications [27]. Yet, some other studies showed that platelet indices were significantly higher in diabetes patients than in control groups [28].

Therefore, it is pertinent to bring to the fore that periodic follow-up and monitoring of changes in haematological parameters are not included in the current guidelines on the management of diabetes, and this could be due to a lack of quality evidence from studies on haematological parameters in diabetes. Perhaps the exception is arguably platelet function among individuals on antiplatelet medicines. This is notwithstanding the fact that HbA1c is a factor in the haemoglobin (Hb) phenomenon. Uncontrolled hyperglycaemia in diabetes over time causes serious destruction of many body systems, especially the blood vessels and nerves. When diabetes is present, the morphology, metabolism, and function of RBC are subject to a series of changes that affect haemorrheology and microcirculation [29]. These changes are due to several factors, including raised levels of ROS and the formation of advanced glycation end products as a result of sustained hyperglycaemia. It has been established that slow glycosylation of RBC is a sensitive index to check body health status [30]. What this review has identified is that it is pertinent to consider changes in other haematological indices in diabetes management.

Haematological Indices and Diabetes Complications

Diabetes has a great link with vascular diseases. The rate of progression of vascular complications in patients with diabetes is more rapid when compared with individuals without diabetes [31]. Both microvascular and macrovascular complications of diabetes are also linked to alterations in coagulation, boosted platelet activation, and abnormal functioning of the endothelium [31]. Some components of MetS and leucocytes showed a link between total WBC and diabetes. Studies have shown that WBC may be associated with T2DM and CHD [32]. Increased differential cell counts of eosinophils, neutrophils, and monocytes indicate a future incidence of CHD [33]. WBC might have a role in the development and progression of diabetic complications [34].

There have been investigations on whether haematological parameters in T2DM can predict microvascular complication development, and the pertinent results are presented in Table 1. There is evidence of agreement from studies regarding the use of WBC differentials to highlight inflammation, platelet indices, haemostasis, and RBC indices to determine anaemia. It has been suggested that the assessment of the neutrophil-to-lymphocyte ratio may be of some benefit in diabetes management [35]. However, there is very limited data on this. Hence, further studies, especially with clinical data, are warranted.

**Table 1:** Haematological parameters in T2DM compared to the control group T2DM: type 2 diabetes mellitus, RBC: red blood cells, WBC: white blood cells, RDW: red cell distribution width, MPV: mean platelet volume, MCV: mean corpuscular volume, MCH: mean corpuscular haemoglobin, MCHC: mean corpuscular haemoglobin concentration, Hb: haemoglobin, HCT: haematocrit

Article	Parameters	T2DM	Control	P < 0.05
Cell counts and RBC indices [22]	WBC (10^3^/μl)	7.01	6.50	<0.05
	Neutrophil (10^3^/μl)	4.14	3.80	<0.03
	Lymphocyte (10^3^/μl)	2.07	1.86	<0.01
	Hb (g/dL)	15.70	16.20	<0.01
	HCT (%)	46.40	46.60	>0.05
	RDW (%)	14.00	13.50	<0.01
	Platelet (10^3^/μl)	262.80	247.30	<0.02
	MPV (fl)	8.50	8.20	<0.02
	MCV (fl)	90.10	90.80	>0.05
	MCH (Pg)	31.00	31.10	>0.05
	MCHC (%)	34.20	34.50	0.05
WBC and platelet counts [35]	Neutrophil (10^3^/μl)	4.68	4.08	<0.02
	Lymphocyte (10^3^/μl)	2.20	7.97	<0.05
	HCT (%)	41.38	41.68	>0.05
	Platelet (10^3^/μl)	279.39	261.62	0.05
	MPV (fl)	8.54	8.53	>0.05

In the study that looked into some WBC and platelet counts [36], the results recommended haematological assessments to predict diabetic microvascular complications, with an emphasis on using a cost-effective and simple blood count (FBC). These results are in agreement with the findings that show statistically significant differences exist between haematological parameters and these haematological indices, and they could be used as an indicator of vascular complications and glycaemic controls in T2DM [37]. Higher levels of differential counts, RDW, platelet count, and MPV were found in T2DM, and these raised values were statistically significant in non-diabetic subjects [23]. Indeed, a study has reported increased RBC fragility during hyperglycaemia due to the competition of glucose entry to RBC with activated ascorbic acid, resulting in a more rigid RBC that is more prone to haemolysis. This is a possible link to the finding of higher RDW [38]. Platelet function is an important pathophysiological factor in the production of atherothrombosis in T2DM. Its activity plays a role in the progression of vascular problems in diabetes. For instance, platelet count and MPV are simple and effective tests that can predict angiopathic or vascular complications in diabetes [39]. Haematological parameters are found to be associated with insulin resistance.

Insulin resistance, on the other hand, is associated with a number of pathologic conditions, such as obesity, T2DM, and chronic inflammation. The interrelationship between insulin resistance and inflammatory activity is complex. It is pertinent to note that WBCs are involved in the release of inflammatory molecules and constitute biomarkers of inflammation not only in CVD but also in T2DM and its complications. Recent studies have reported a decrease in red cells and their lifespan in T2DM due to elevated blood glucose [40].

Lipid Profiles and Diabetes Mellitus

There has been evidence of a correlation between blood glucose levels and serum lipid profiles [41]. This includes negative and positive correlations of glycaemia with HDL and TC, respectively [42]. Dyslipidaemia and hypertension are modifiable risk factors for T2DM and related CVD and account for disability in more than 87% of the LMIC [43]. Understanding the relationship between serum lipid patterns and different stages of glucose intolerance is useful in clinical and public health, as such data can potentially form the basis for future preventive programs for diabetes and related complications. This study will look at lipid levels and the prevalence of lipid abnormalities during diabetic progression. Dyslipidaemia is an established risk factor for T2DM, and together with hypertension, insulin resistance, and obesity, it constitutes the metabolic disarray that could cause T2DM [44].

The lipid abnormalities found in T2DM are also found in prediabetics, and the detection and correction of lipoprotein abnormalities in patients with diabetes are important to prevent pancreatitis due to severely high levels of TG and to reduce the risk of macrovascular complications.

Current guidelines for the management of diabetes recommend that patients be tested for blood lipid profiles at the time of diagnosis and then at least once every year [45]. The idea is to achieve levels of HbA1c ≤6.5% as well as LDLC <3.0 mmol/l and HDLC >1.0 mmol/l in diabetes under treatment. Abnormal levels can, therefore, be used as a possible biomarker in predicting the risk of dyslipidaemia and, consequently, the risk of developing CVD in diabetic patients [46]. Good glycemic control has a favorable effect on lipoprotein levels in diabetes with a reduction in cholesterol and TG levels by lowering circulating VLDLC and increasing the catabolism of LDLC through reduced glycation and upregulation of the LDLC receptors [47].

Studies have suggested monitoring Hb and HbA1c alongside dyslipidaemia as part of preventive early identification and management of gestational diabetes mellitus [42]. Others indicated that improved glycaemic control indicated by lowered HbA1c has a beneficial effect on the lipid profiles of patients, while other studies showed either no considerable relationship or a negative relationship among the abovementioned parameters [48,49].

Despite these established principles in clinical practice, it is important to note that disparities still exist in the affordances (i.e. accessibility and affordability) of lipid profile tests among the LMIC [50-52]. This limited affordability factor can potentially impact diabetes management, hence the need for consideration of cost-effective routine haematological measurements.

Dyslipidaemia and Diabetes Complications

Diabetes mellitus is the most common metabolic disorder, affecting people worldwide [53]. T2DM is caused by interactions between the environment and genetic factors [31]. Individuals with T2DM have a two- to fourfold increased risk of CHD, which is the leading cause of death among people with T2DM [54], and a significant part of this risk is attributed to diabetic dyslipidaemia [55]. Long-term complications of poor glycemic control contribute significantly to the morbidity, mortality, and economic burden of diabetes [56]. Microvascular complications of diabetes are found in the kidneys, retina, and peripheral nerves, resulting in nephropathy, retinopathy, and neuropathy, respectively, while macrovascular complications cause accelerated atherosclerosis, increasing the risk of myocardial infarction and stroke [46].

Lipid abnormalities are common in people with diabetes and prediabetes [57], but the pattern of different lipids may show variations among ethnic groups, economic levels, and people's access to health care [58]. New discoveries have provided hope to minimize the morbidity and mortality of T2DM, which is one of the most common causes of hyperlipidemia [53].

The relationship between hyperlipidaemia and vascular complications of diabetes has long been of interest because both tend to occur with greater frequency in T2DM [55]. Diabetes initiates and is responsible for microvascular lesions, and it is believed that insufficient control of diabetes accentuates microangiopathy. In fact, optimal control of diabetes should achieve normal blood glucose and a normal level of HbA1c, as well as the absence of reversible accompanying complications such as hyperlipidaemia, red cell rigidity (i.e. haematology), and increased capillary permeability [59]. Although there is a dearth of studies on the possible correlation between haematological parameters and lipid profile indices, a study has indicated some interesting correlation (R values) results (Table 2).

**Table 2:** Reported correlation between haematological parameters and lipid profiles TC: total cholesterol, RBC: red blood cells, HDL: high-density lipoprotein, LDL: low-density lipoprotein, TG: triglycerides, Hb: haemoglobin, HCT: haematocrit [10]

Parameter	TC	HDL	LDL	TG
Platelets	-0.496	-0.478	-0.522	-0.488
RBC	0.526	0.498	0.532	0.518
Hb	-0.514	-0.510	-0.518	-0.499
HCT	-0.525	-0.521	-0.530	-0.518
Lymphocytes	0.432	0.552	0.401	0.512

Diabetes affects metabolism as well as every cell of the body; hence, it is associated with many complications [60]. Studies have put the incidence of dyslipidaemia in diabetes at between 21% and 81% [61]. Suffice to bring to the fore that the pathophysiology of insulin resistance in T2DM involves complex metabolic abnormalities [44], which include but are not limited to increased release of free fatty acid from the adipocytes and the underlying cause of dyslipidaemia [46]. The cluster of interrelated lipoprotein abnormalities and plasma lipid abnormalities is closely related to each other. A characteristic pattern referred to as diabetic dyslipidaemia comprises reduced levels of HDL, increased TG, and excess small LDL [44].

Additionally, there is the concept of higher TG-rich lipoproteins (TGRL) such as VLDL, resulting in the exchange of TG and cholesterol between TGRL and LDLC and HDLC. As such, one of the hallmarks of insulin resistance is the smaller particles of HDLC and LDLC. Significantly, while small LDLC is extremely atherogenic, small HDLC is also dysfunctional and can be lost renally [62,63]. This pattern, usually seen in T2DM, may be a treatable risk factor for CVD [44]. Dyslipidaemia is known to influence the haematopoietic process, especially the WBC. The main reason for the management of dyslipidaemia is to reduce the levels of LDLC and other apolipoprotein-B-containing lipoproteins [64]. Given the potential impact on haematology, it is necessary to establish the level of relationship between haematological parameters and lipid profiles in T2DM.

Review of correlation between haematological parameters and lipid profiles

This followed a systematic research approach. Article search platforms were discretionally limited to two (Google Scholar and PubMed) to minimize duplicates. The Google Scholar search included the correlation between haematological parameters and lipid profiles in diabetes, limited to listed titles on pages 1 to 3 (sorted by relevance). The PubMed search followed a sequence of 'diabetes mellitus//lipid profile//haematology//correlation', limited to five years, and free full-text articles.

Results of the Literature Search

**Figure 2:**
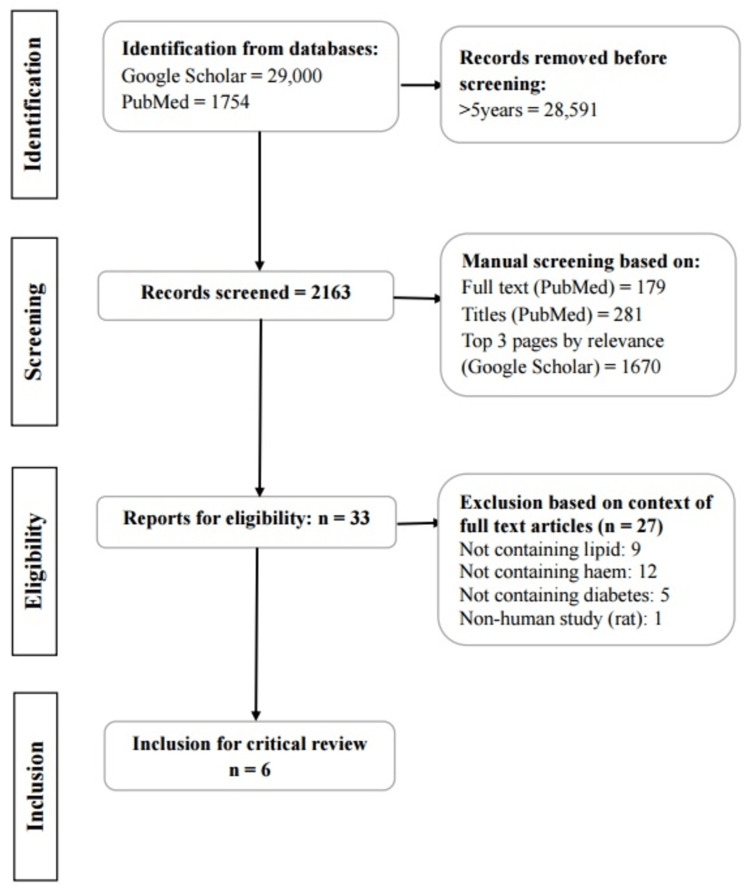
PRISMA flowchart of literature search PRISMA: Preferred Reporting Items for Systematic Reviews and Meta-Analyses

The PubMed search with all key terms yielded 1,754 articles, but only three were selected after all exclusions [65-67]. The Google Scholar search yielded over 29,000 articles, but only 30 were selected for review after limitations. Figure 2 shows that among the 33 articles assessed, only six were selected and critically reviewed [6,10,30,65-67].

Mixed Correlation Between Haematological Parameters and Lipid Profiles

Abnormalities in patients with T2DM: In a study in a population of 304 patients with T2DM, a lipid profile was done to determine the level of TG, TC, HDLC, and LDLC. Haematological parameters were also determined by FBC, consisting of RBC, Hb, total WBC, differential count, platelets, and Hb indices [10]. Pearson-moment r correlation was used to investigate the presence of any significant relationship between haematological parameters and lipid profiles among diabetic patients. The finding shows a strong positive correlation between RBC, lymphocytes, and different lipid profiles, which was also significant (p = 0.003 and p = 0.002, respectively). However, there was a strong negative correlation between platelets, haematocrit (HCT), and Hb (Table 2).

At baseline moderate anaemia: In a prospective cohort study involving 30 patients with T2DM and 25 age-matched apparently healthy individuals as controls, the study investigated various haematological parameters and lipid profiles; at baseline moderate anaemia, WBC and basophil were significantly higher in diabetic subjects compared to control subjects [30]. Table 3 shows that baseline results show expected higher values in diabetes mellitus compared to the healthy group (Table 3). Also, Pearson correlation highlighted a strong positive correlation between basophils and TC, which was significant (p = 0.000), as well as a strong negative significant correlation between WBC, monocytes, eosinophils, Hb, mean corpuscular haemoglobin (MCH), mean corpuscular volume (MCV), packed cell volume (PCV), platelet count, and TC. WBC, lymphocytes, MCV, and platelet count correlated negatively with HDL, among others (Table 4).

**Table 3:** Baseline values of haematological and lipid levels in T2DM vs. control T2DM: type 2 diabetes mellitus, WBC: white blood cell, HDL: high-density lipoprotein, TG: triglycerides

Parameters	Diabetes	Non-diabetes
WBC count (x 10⁹/L)	5.86	3.82
Basophil (%)	0.52	0.32
HDL (mmol/L)	1.2	1.99
TG (mmol/L)	1.74	1.46

**Table 4:** P-values of Pearson correlation haematological parameters and lipid profiles in diabetes mellitus TWBC: total white blood cells, RBC: red blood cells, Hb: haemoglobin, PCV: packed cell volume, MCV: mean corpuscular volume, MCH: mean corpuscular haemoglobin, MCHC: mean corpuscular haemoglobin concentration, TC: total cholesterol, HDL: high-density lipoprotein, LDL: low-density lipoprotein, VLDL: very low-density lipoprotein, TG: triglycerides *six months of prospective follow-up and statistical significance, **high to very high statistical significance

Parameters	TC		HDL		LDL		TG		VLDL	
	Baseline	6 months*	Baseline	6 months*	Baseline	6 months*	Baseline	6 months*	Baseline	6 months*
TWBC	0.000**	0.034*	0.000**	0.421	0.000**	0.361	0.000**	0.702	0.000**	0.398
Neutrophils	0.498	0.005**	0.003**	0.699	0.027*	0.019*	0.766	0.872	0.667	0.753
Lymphocytes	0.091	0.021*	0.000**	0.601	0.000**	0.197	0.315	0.88	0.36	0.553
Monocytes	0.001**	0.762	0.000**	0.047*	0.000**	0.12	0.000**	0.717	0.000**	0.086
Eosinophils	0.007**	0.179	0.000**	0.141	0.000**	0.036	0.001**	0.721	0.001**	0.076
Basophils	0.000**	0.242	0.000**	0.025*	0.362	0.013*	0.001**	0.86	0.014*	0.056
RBC	0.44	0.282	0.001**	0.173	0.012*	0.983	0.885	0.434	0.789	0.002**
Hb	0.004**	0.927	0.001**	0.691	0.000**	0.835	0.049*	0.984	0.065	0.000**
PCV	0.003**	0.902	0.11	0.764	0.000**	0.907	0.073	0.999	0.104	0.000**
MCV	0.000**	0.052	0.000*	0.007**	0.148	0.923	0.000**	0.141	0.000**	0.693
MCH	0.000**	0.018*	0.629	0.099	0.000*	0.43	0.000**	0.123	0.000**	0.56
MCHC	0.813	0.637	0.000**	0.573	0.009**	0.957	0.606	0.679	0.52	0.885
Platelet count	0.003**	0.776	0.003**	0.002**	0.461	0.019*	0.461	0.633	0.073	0.041*

Comparison of Diabetes and Non-diabetes for Key Haematological Parameters and Lipid Profiles

A brief critical review of the comparison of diabetic and non-diabetic overweight subjects for key parameters of blood biochemistry and haematology was done, where blood was analyzed and data recorded [6]. The data consisted of measured concentrations of biochemical markers (TC, LDLC, HDLC, and TG) and haematological parameters (Hb, HCT, RBC, and WBC). Statistically significant results were obtained for TC (p = 0.01, LDLC p < 0.001, TG p < 0.001) between diabetics and non-diabetics, while no statistically significant result was obtained for HDLC p > 0.05. For haematological parameters, RBC, Hb, and HCT showed significant differences in women but not in men. The gender differences in the levels of erythrocytes, Hb, and HCT could create a need for further studies in this area (Table 5).

**Table 5:** Haematological parameters and lipid profiles in diabetes vs. non-diabetes overweight subjects TC: total cholesterol, LDLC: low-density lipoprotein cholesterol, TG: triglycerides, Hb: haemoglobin, HCT: haematocrit

Parameters		Diabetes	Non-diabetes	Statistical significance
TC (mmol/L)		10.78	12.19	Yes
LDLC (mmol/L)		5.94	7.26	Yes
TG (mmol/L)		8.70	11.42	Yes
Erythrocytes (10³/μl)	Women	4.00	4.60	Yes
	Men	4.10	5.10	No
Hb (mg/dl)	Women	12.00	13.60	Yes
	Men	14.00	7.80	No
HCT (%)	Women	37.00	40.10	Yes
	Men	40.00	31.10	No

RDW and Glycaemic Index in T2DM

In a study of the association of RDW with glycemic index in T2DM [65], 130 individuals admitted to a diabetic center were divided into two groups: prediabetic and diabetic, with 65 healthy people as controls. Lipid profiles and FBC were determined for each participant, and differences in those parameters between groups were evaluated using a one-way ANOVA test. Significantly higher levels of cholesterol, TG, and LDL were reported in prediabetic and diabetic patients than in the normal control group (p < 0.001, p < 0.001, and p < 0.001, respectively). HDL was significantly lower in both prediabetic and diabetic patients than in the control group. CBC performed to investigate anaemic status in the prediabetic and diabetic patients showed no anaemia, and no significant differences were observed in RBC, Hb, and HCT for both prediabetic and diabetic patients compared to controls (p = 0.975, p = 0.175, and p = 0.393, respectively) (Table 6).

**Table 6:** Haematological parameter and lipid profile correlation of changes in diabetes RDW: red cell distribution width, TG: triglycerides, LDLC: low-density lipoprotein cholesterol, HDLC: high-density lipoprotein cholesterol

Parameter	Control	Prediabetic	Diabetic	Comment
RDW (%)	12.7	13.5	15.2	Significant
Cholesterol (mmol/L)	6.64	8.25	13.71	Significant
TG (mmol/L)	5.44	7.73	12.62	Significant
LDLC (mmol/L)	4.97	5.51	7.44	Significant
HDLC (mmol/L)	2.78	2.36	2.13	Significant

This report was in agreement with the findings of [30]. RBC, HB, and HCT showed no significant difference but disagreed with the report that showed HB being significantly lower in T2DM compared to control groups [23]. These inconsistencies in haematological parameters and lipid profiles necessitate further studies to unravel some hidden facts about diabetes.

MPV and MetS in T2DM

The association between MPV and MetS in patients with T2DM was investigated [66]. About 1,240 patients with T2DM were enrolled in this study, of whom 873 had MetS. Lipid profiles and FBC data were collected retrospectively via medical records. MPV was reported to be significantly higher in patients with MetS (p < 0.001). For individual MetS components, MPV was significantly higher in abdominal obesity and hypertriglyceridaemia (p = 0.013 and p = 0.026, respectively) but did not differ in the presence of low HDLC (p = 0.790). MPV was independently associated with MetS after adjustment for sex, smoking, alcohol drinking, and WBC. In a stratified analysis, the positive correlation of MPV with MetS was significant only in patients who were older, male, or overweight or had poor glycaemic control (Table 7).

**Table 7:** Haematological parameters and lipid profiles in MetS MetS: metabolic syndrome, WBC: white blood cell, MPV: mean platelet volume, TG: triglycerides, HDLC: high-density lipoprotein cholesterol

Variables	MetS	No MetS
WBC count (x10⁹/L)	6.93	7.38
MPV (Fl)	8.81	9.34
TG (mmol/L)	1.16	2.21
HDLC (mmol/L)	1.22	0.97

Monocyte-to-HDL Ratio and Arterial Stiffness in Diabetes

The association of monocyte-to-HDL ratio (MHR) with arterial stiffness in patients with diabetes was examined [67]. Eighty-one patients with diabetes participated in this cross-sectional study. Cardio-ankle vascular index (CAVI) was used as an index of arterial stiffness. FBC and lipid profiles were conducted for all the participants, and statistical analysis was performed to determine the relationship between MHR and CAVI. Receiver operating characteristic analysis was used to estimate the cut-off values on MHR to predict CAVI ≥ 9.

The authors found that the mean of CAVI increased with age and was higher in males. Spearman analysis showed a significant positive correlation between MHR and CAVI (ρ = 0.239 and p = 0.031, respectively). The correlation between CAVI and HDL cholesterol levels was negative (p = -0.284 and p = 0.01, respectively). There was no correlation between CAVI and monocyte count (p = 0.156 and p = 0.165, respectively). MHR and HBA1c were independently associated with CAVI. The authors reported that, besides HbA1c, only MHR showed a statistically significant association with CAVI, whereas no other traditional risk factor showed a significant association at p < 0.05 (Table 8).

**Table 8:** Relationship between traditional risk factors of cardiac event and CAVI CAVI: cardio-ankle vascular index, BMI: body mass index, HbA1c: haemoglobin A1C, LDLC: low-density lipoprotein cholesterol, MHR: monocyte-to-high-density lipoprotein ratio, TC: total cholesterol

Index	Odds ratio	p < 0.05
BMI	0.97	No
Gender	0.44	No
HbA1c	1.29	Yes
Hypertension	1.43	No
LDLC	1	No
MHR	1.14	Yes
Smoking	4.36	No
TC	1.01	No

Discussion and narrative summary

Diabetic mellitus is a metabolic disease characterized by hyperglycaemia, caused by disorders of carbohydrate metabolism resulting from insulin deficiency due to chronic insulin resistance, beta cell exhaustion, or an autoimmune process (T2DM vs. T1DM). Although there are disorders of secretion, these are monogenic and very rare. Two main risk factors are genetic and environmental factors. Insulin resistance is a major factor in the pathogenesis of diabetes and depends on diet, which, if improperly managed, may lead to disruption of lipid profiles. The result of this review demonstrates that alteration of haematological parameters and lipid profiles occurs during diabetes but is not consistent. Perhaps it is pertinent to highlight that not all studies observe a negative or positive correlation [68].

In the first reviewed article, Antwi-Baffour et al. showed that all lipid profiles except TC were significantly higher in males than females [10]. TC levels were higher in females compared to males. With HDL, there is a report of no statistically significant difference between gender groups [6], whereas in the report from Nigeria, significantly higher HDL in normal subjects but not in TC was the finding [30]. Indeed, there is concern about the application of gender-different cutoffs being able to confound research findings [69], and this is with cognizance of other reports on gender differences in TC [70,71]. Thus, there are variations in reports, which can be explained by at least two factors: differences in research objectives and population.

The latter, population, is quite an important point of note, especially in countries like Australia, which is fast growing in multiethnicity. For instance, Africans are known to be genetically disposed to low HDL compared to Europeans [72]. Nevertheless, in terms of research objectives, only one of the studies is longitudinal [30]. It is still important to do further studies to investigate longitudinally whether lipid profile indices differ between gender groups as well as with changes in HbA1c and correlations with haematological parameters among individuals living with diabetes.

The results showed mixed correlations. TC correlated positively with basophils, while it correlated negatively with WBC, monocytes, eosinophils, Hb, PCV, MCV, MCH, and platelets [30]. This negative correlation of TC with platelets, Hb, and PCV agreed with the findings of Antwi-Baffour et al. [10]. HDL correlated positively with neutrophils, monocytes, basophils, Hb, and RBC and negatively with WBC, lymphocytes, and platelets. More interesting in this report is the correlation and level of significance achieved between haematological parameters and lipid profiles and the changes observed after six months in the second study. For instance, WBC showed a correlation with significance across all the lipid profiles at baseline, but after six months, the significance level changed across all the lipid profiles, and WBC showed significance with only TC.

This pattern of change was also found for monocytes, where significance was found only with HDL after six months. Other haematological parameters and lipid profiles showed this varied significance of correlation, as can be seen in Table 2. This result came from a prospective cohort study using data recorded over six months. To grasp a deeper insight into these observed changes, the present research will adopt a retrospective study using data generated over 12 months to look at these correlations and changes in significance. The authors reported higher levels of cholesterol, TG, and LDL in the diabetic and prediabetic groups, while HDL was significantly lower in both groups [65]. Only the RDW of all the measured haematological parameters showed a statistically significant difference between the three groups. The author reported TC, LDL, and TG being significantly different from the control, while haematological parameters show gender differences, where HCT, Hb, and RBC were significant only in females [6].

With a special interest in platelet indices in MetS [66], another study reported MPV was significantly higher in obesity and hypertriglyceridaemia. MPV and WBC were the only haematological parameters that showed significant differences, while TG and HDL were lipid profiles that showed statistical differences from the control. CAVI is a useful index of arterial stiffness and is associated with MHR in patients with diabetes, irrespective of various potential confounders. This agrees with the earlier report that showed a positive and statistical correlation of MHR with arterial stiffness in patients with untreated hypertension [73].

## Conclusions

This literature review has assessed the changes in haematological parameters and lipid profiles associated with diabetes. The review also determined the potential correlations between haematological parameters (full blood count) and lipid profiles. There is knowledge that dyslipidaemia, but not an abnormal haematological parameter, is established to be associated with diabetes. Hence, periodic monitoring of lipid profile changes but not routine haematological parameters are included in the current guidelines on the management of diabetes. Yet, the issue of availability and cost of lipid profile tests in LMIC could be addressed by correlating haematological parameters to diabetes. The findings from this review show that many studies have looked at haematological parameters and lipid profiles in diabetes separately, but very few have investigated their concordance. Based on the literature that was critically reviewed, there is evidence of haematological indices being correlated with dyslipidaemia in diabetes, but some inconsistencies in the correlation warrant further studies. The significance lies in the potential for utilizing routinely available clinical evaluations of blood cell indices in diabetes management and its complications. Further, there is a dearth of longitudinal studies; hence, a retrospective analysis would be beneficial to investigating the correlation. This is important to advance the justification for the use of more commonly available routine haematological parameters for predicting and managing diabetic complications.
